# Intra-accumbens Raclopride Administration Prevents Behavioral Changes Induced by Intermittent Access to Sucrose Solution

**DOI:** 10.3389/fnins.2018.00074

**Published:** 2018-02-21

**Authors:** Josué O. Suárez-Ortiz, Felipe Cortés-Salazar, Ariadna L. Malagón-Carrillo, Verónica E. López-Alonso, Juan M. Mancilla-Díaz, Juan G. Tejas-Juárez, Rodrigo E. Escartín-Pérez

**Affiliations:** ^1^Laboratory of Neurobiology of Eating, Facultad de Estudios Superiores Iztacala, Universidad Nacional Autónoma de México, Tlalnepantla, Mexico; ^2^División Académica Multidisciplinaria de Comalcalco, Universidad Juárez Autónoma de Tabasco, Tabasco, Mexico

**Keywords:** binge-type eating, dopamine d2 receptors, microstructure, raclopride, intermittent sucrose, nucleus accumbens shell

## Abstract

Overeating is one of the most relevant clinical features in Binge Eating Disorder and in some obesity patients. According to several studies, alterations in the mesolimbic dopaminergic transmission produced by non-homeostatic feeding behavior may be associated with changes in the reward system similar to those produced by drugs of abuse. Although it is known that binge-eating is related with changes in dopaminergic transmission mediated by D2 receptors in the nucleus accumbens shell (NAcS), it has not been determined whether these receptors may be a potential target for the treatment of eating pathology with binge-eating. Accordingly, the aim of the present study was to evaluate whether sugar binging induced by intermittent access to a sucrose solution produced changes in the structure of feeding behavior and whether blocking D2 receptors prevented these changes. We used the intermittent access model to a 10% sucrose solution (2 h/day for 4 weeks) to induce sugar binging in Sprague Dawley female rats. Experimental subjects consumed in a 2-h period more than 50% of the caloric intake consumed by the subjects with ad-lib access to the sweetened solution without any increase in body weight or fat accumulation. Furthermore, we evaluated whether sugar binging was associated to the estrous cycle and we did not find differences in caloric intake (estrous vs. diestrus). Subsequently, we characterized the structure of feeding behavior (microstructural analysis) and the motivation for palatable food (breakpoints) of the subjects with sugar binging and found that feeding episodes had short latencies, high frequencies, as well as short durations and inter-episode intervals. The intermittent access model did not increase breakpoints, as occurred in subjects with ad-lib access to the sucrose. Finally, we evaluated the effects of D2 receptor blockade in the NAcS, and found that raclopride (18 nM) prevented the observed changes in the frequency and duration of episodes induced by intermittent access to the sucrose solution. Our results suggest that alterations in behavioral patterns associated with binge-eating behavior depend in part on the dopaminergic transmission in the NAcS and that the antagonism of D2 receptors may be a therapeutic tool for feeding pathology with binge-eating.

## Introduction

Excessive consumption of highly-palatable and energy-dense foods is frequently found in patients with eating disorders (binge eating disorder, BED, and bulimia nervosa, BN) and in some obese patients (Davis, 2017). Specifically, in individuals diagnosed with BED and BN, binging is a key eating disorder feature, and the energy restriction is not a necessary condition (DSM-5, APA, 2013). On the other hand, obesity is a multifactorial entity, which notably includes prominent increase of energy intake, promoting unhealthy accumulation of adipose tissue (WHO, 2016), and binging episodes are frequently present (Burrows et al., 2017). Despite the fact that the triggering factors of binging in those pathological conditions are different, similarities in symptomatology (consumption of large amounts of food, reduced control over eating and continued overconsumption of food despite negative consequences, and impulsivity; APA, 2013; Pivarunas and Conner, 2015), strongly suggest overlapping of the neurobiological factors involved (Davis, 2017), and such factors correspond with those described in the development of the addictive processes. According to clinical evidence, almost 57% of overweight/obese adults diagnosed with BED met the diagnostic criteria for food addiction (Gearhardt et al., 2012).

The food addiction construct is still a matter of debate, but there is some evidence that strongly suggest that genetic variations (multilocus genetic profile, MLGP) related with enhanced dopamine signaling (involving functional changes in D2 receptors, dopamine transporter, and Catechol-ortho-methyltransferase) may lead to elevated responsiveness to palatable food and high scores in the YFAS (Yale Food Addiction Scale) (Davis et al., 2013). Moreover, a pattern of reward-related activation in brain regions associated with substance dependence (including the anterior cingulate cortex, left medial orbitofrontal cortex, and left amygdala) showed positive correlations with YFAS scores (Gearhardt et al., 2011). Together, these findings support the idea that altered dopamine transmission is one of the main neurobiological factors that explain the high comorbidity between BED and obesity.

Another similarity between the symptomatology of patients with BED and with obesity is that most of the binging episodes include food with high-sugar content. In this regard, it has been shown experimentally that chronic consumption of these palatable diets produces changes in circuits that regulate the expression of satiety (reducing the activity of the anorexigenic system of oxytocin) (Mitra et al., 2010). Furthermore, neuroimaging studies have shown that patients with BED have greater activation of the orbitofrontal cortex and enhanced reward sensitivity while viewing images of hypercaloric foods (Schienle et al., 2009). Correspondingly, it seems plausible that reduction in central satiety signaling (Mitra et al., 2010) and changes in the reward system caused by chronic intake of palatable high-sugar foods (Cooper, 2004) may lead or preserve binging behavior.

Clinical and experimental evidence confirms that binging behavior is associated with alterations on dopaminergic transmission (Bello and Hajnal, 2010). For instance, experiments with rats have shown persistent increase in extracellular DA in the nucleus accumbens shell (NAcS) following repeated and intermittent intake of palatable solutions (10–25% sucrose), modifying the mesolimbic dopaminergic system (Rada et al., 2005), and producing a pattern of excessive intake as well as increments in μ-opioid and D1 dopamine receptor binding (Colantuoni et al., 2001), similar to repeated administration of a drug of abuse (Avena et al., 2008a).

Interestingly, it has been observed that obesity-prone rats have impairments in the mesoaccumbal dopamine system, including the reduction in basal and electrically-stimulated dopamine release (Geiger et al., 2008), as well as reduced dopamine release induced by direct intragastric injection of a lipid emulsion that increases triglycerides levels (Rada et al., 2010). This hypodopaminergic profile leads to hyperphagia as a compensatory response to elevate central dopamine release; however, the mesolimbic reward circuitry is less sensitive to general reward but remains responsive to palatable foods (Geiger et al., 2009; Leigh and Morris, 2018). In line with these findings, it was reported that chronic exposition to a palatable energy-dense diet induces overweight and downregulation of striatal dopamine D2 receptor expression, and lentivirus-mediated knockdown of striatal D2 receptors in the dorsolateral striatum, causes compulsive-like feeding behavior and reward hypofunction in rats exposed to the palatable energy-dense diet (Johnson and Kenny, 2010). Human brain imaging data extend these findings since it was observed a blunted amphetamine-induced dopamine release and lower baseline striatal dopamine D_2/3_ receptor availability in obese compared to normal-weight women (van de Giessen et al., 2014).

D2 dopamine receptors have received special attention in clinical and experimental research of pathologies with binge-eating behavior. Since D2 receptors in the NAcS play a key role as mediators of the regulation of rewarding properties of pleasurable stimuli (Hajnal et al., 2008), Davis et al. (2012), examined genetic markers for increased dopaminergic activity and changes in expression of D2 receptors (single nucleotide polymorphisms rs1800497, rs1799732, rs2283265, rs12364283, and rs6277) in BED patients. They found that a greater proportion of BED participants were significantly related to genotypes (rs1800497 and rs6277) that reflect enhanced dopaminergic neurotransmission and were less likely to carry the minor T allele of rs2283265, changing the relative equilibrium expression between short and long isoforms of the D2 dopamine receptors (DRD2S and DRD2L, short and long isoforms, respectively) toward DRD2S and presumably increasing the activity of the striatum and the prefrontal cortex (Zhang et al., 2007). Furthermore, experimental evidence of animal models has revealed that sugar binging is related to changes in dopaminergic activity mediated by D2 receptors: decreasing D2 receptor binding in the striatum (Colantuoni et al., 2001; Bello et al., 2002) and D2 receptor mRNA expression in the nucleus accumbens (Spangler et al., 2004).

Moreover, D2 dopamine receptors have been implicated in the susceptibility for reward seeking behaviors including predisposition to diet-induced obesity (with high-sugar palatable diets) and drug addiction, probably through a mechanism of sensitization of the reward system in which the signaling of the D2 receptors is altered by the sustained increase of the release of dopamine in the NAcS (Hajnal et al., 2008). Accordingly, peripheral blockade of D2 receptors in male rats decreased sugar intake with intermittent or free access to sucrose solutions (Corwin and Wojnicki, 2009).

Binge-eating behavior is well-defined clinically and experimentally (Corwin et al., 2011; APA, 2013); however, little information exists on the behavioral characterization of the microstructure of feeding behavior in animals that express sugar-induced binging. Furthermore, although animal studies and clinical binge-eating research have reported specific changes in the dopaminergic transmission mediated by D2 receptors in the NAcS, it has not been determined whether these receptors are a potential target for the treatment of eating pathology with binging. Following this line of reasoning, the aim of the present study was to evaluate the motivation for palatable food (breakpoints), to characterize the structure of feeding behavior (microstructural analysis) of female rats with sugar binging induced by intermittent access to a sucrose solution (10%), as well as to evaluate whether blocking D2 receptors in the NAcS prevented these changes. In this study, female rats were chosen since feeding behavior is clearly influenced by estradiol (Eckel, 2011), and it has been observed that female rats are susceptible to develop sugar binging (Klump et al., 2011; Calvez and Timofeeva, 2016) and escalate caloric intake faster than male rats (Babbs et al., 2011).

## Materials and methods

### Subjects

Female Sprague Dawley rats (250–300 g, Proyecto Camina, A.C., Mexico) were individually housed and maintained under an inverted 12-h light/dark cycle (lights on at 17:00 h) and at 22 ± 2°C. Water and standard rodent diet (LabDiet® formulab diet #5008) were provided *ad libitum* unless otherwise specified (lever pressing training). All experimental procedures were carried out in accordance with the *Norma Oficial Mexicana Para el Cuidado y Uso de Animales de Laboratorio* (NOM-062-ZOO-1999) and the protocol was approved by the FES Iztacala Ethics Committee. The present study was designed to use the minimum number of rats. Those that were subject to stereotaxic procedures were allowed to recover from surgery in their home cages for 5 days before experimental procedures began.

### Drugs

Raclopride tartrate salt (D2 receptor antagonist) (Sigma Chemical Co., Toluca, Mexico) was dissolved in 0.9% saline solution. The drug was prepared freshly at the appropriate concentration just before administration.

### Determination of the estrous cycle phases

Estrous phase was determined by the relative number of cells in vaginal smears dyed with cresyl violet and examined under light microscopy (predominance of nucleated epithelial cells, proestrus; anucleated cornified cells, estrous; the same proportion among leukocytes, cornified, and nucleated epithelial cells, metestrus; predominance of leukocytes, diestrus). The estrous phase was determined before the beginning of the exposure to the sucrose solution (intermittent/*ad libitum*) in order to synchronize the beginning of the experimental protocol to the middle of diestrus.

### Intermittent access to sucrose protocol

Subjects were matched by weight and were assigned to three independent groups and differentially exposed to a sucrose solution (10% w/v): no access (control, *n* = 14), 2-h daily access (intermittent, *n* = 16) and 24-h daily access (*ad libitum, n* = 13) during 28 days with free access to standard chow and tap water. Those groups exposed to sucrose (intermittent/*ad libitum*) had simultaneously sucrose solution and tap water in separated bottles. All animals started this protocol in the middle of the diestrus phase. Food and sucrose solution intake, as well as the estrous phase, were evaluated on a daily basis. Body weight was monitored once a week during the entire protocol. The calorie content of the 10% sucrose solution was 0.4 kcal/ml (chow was 3.56 kcal/g). Food and sucrose solution intake were measured in grams and converted to kcal.

### Measure of adipose tissue

At the end of the behavioral test and after 4 weeks of the intermittent access to sucrose protocol, 4 subjects of each group (control, intermittent and *ad libitum*) were euthanized with an overdose of sodium pentobarbital. Subsequently, the mesenteric, gonadal, inguinal, retroperitoneal, perirenal, interscapular brown adipose tissue (IBAT) and total adipose tissue were carefully dissected. Once extracted, every tissue was weighed individually, according to the method used by Ravagnani et al. (2012) and Mann et al. (2014). These measures were expressed as the normalization of mg of adipose tissue/100 g of body weight.

### Operant evaluations

#### Lever pressing training

Independent rats were divided in three groups (control, intermittent, and *ad libitum, n* = 5 each group). Prior to the intermittent access to the sucrose solution protocol, they were trained to press a lever in exchange for a sucrose reward tablet (45 mg pellets, chocolate flavor, 5TUT Test Diet St. Louis, MO, USA) in 2 sessions of 30 min each, under a variable-interval (VI) 60 s schedule of reinforcement. For the following 7 days, the rats were trained using different fixed-ratio (FR) schedules (days 1–5, FR1; days 6–9 FR5), and these sessions finished when the subject obtained 30 rewards or after 30 min (modified from Floresco and McLaughlin, 2008). Subsequently, breakpoints (BP) were obtained using a progressive ratio (RP) schedule of reinforcement with increasing response requirements according to the equation *response ratio* = [5e^(0.2 × trial number)^] − 5 (Richardson and Roberts, 1996). The PR schedule sessions finished when the subject failed to obtain at least one reward in 60 min or in absence of responses in a 4-min period. The ratio completed (last reward obtained) before the end of the session was considered as BP. Rats were always placed in the operant chambers at the same time of day and they were food-deprived during the first 4 h of the beginning of the dark photoperiod (only in FR sessions).

#### Evaluation of motivation for palatable food

Once the intermittent access to sucrose protocol was completed, all subjects (control, intermittent and *ad libitum* groups) returned to the standard chow diet (sucrose solution-free) and BP were obtained through daily PR sessions. Evaluation of motivation for palatable food finished when three consecutive sessions showed a maximum of 20% of variation relative to the mean ratio response of each subject (in the Results section, these data are expressed as the mean of the last three sessions). Rats were not food-deprived before the session, and tested at same time the intermittent group had access to the sucrose solution.

### Apparatus

We used standard operant chambers (MED Associates, St. Albans, VT, USA) enclosed in sound-attenuating boxes, with two retractable levers (one located on each side of a central food receptacle where food reward pellets were delivered), and illuminated by a 100-mA house light located on the wall opposite the levers.

### Microstructure of feeding behavior

Subjects were video-recorded (one continuous 60-min recording) and the recording was subsequently processed to compute the parameters of meal frequency, meal duration (s), inter-meal interval (s), latency (s), local eating rate (kcal/duration), and durations (s) of activity (sniffing, rearing, grooming, locomotion, and non-ambulatory movements) and resting (inactivity with or without closed eyes, with the head of the rat in the floor of the home-cage). At the end of the observation period, standard chow and sucrose solution consumptions were measured and converted to kcal.

### Stereotaxic surgery

Rats were deeply anesthetized with ketamine/xylazine (112.5/22.5 mg/kg i.p.), and stainless steel injection cannulas (23 G, 15 mm length; Becton Dickinson and Co., Mexico) were stereotaxically implanted in the right NAcS (AP +1.5 mm, ML −1.2 mm, DV −6.0 mm relative to bregma) (Paxinos and Watson, 1998). All subjects were treated post-surgery with enrofloxacin (2.5 mg/kg i.m.) in order to prevent infections. This independent group of rats (*n* = 8) was exposed to the intermittent access to the sucrose solution protocol (28 days) before the surgery. Subsequently, the rats had a recovery period with water and food *ad libitum* (5 days) and then they were re-inserted to the intermittent access to the sucrose solution until they returned to a stable consumption (similar to the consumption at the end of the 28th day of the intermittent sucrose protocol for at least 3 consecutive days).

### Drug injections

Following the recovery period and the re-insertion to the intermittent access to the sucrose, the rats received a mock injection. The following day they were randomly assigned to one of two conditions, and injections of vehicle or raclopride 18 nM (*n* = 8) were applied intra-NAcS 3.5 h after the onset of the dark phase of the light cycle (2 days later they received the opposite treatment, in this counterbalanced design each subject received 2 injections with 1 day of separation between injections). The intra-NAcS treatments were infused in a volume of 0.5 μl at 0.25 μl/min with a 31 G injector that extended 0.3 mm beyond the guide cannula, and the injector remained 1 additional min after the injection to guarantee diffusion of the solutions. After the microinjections, rats were placed into their home cages with pre-weighted standard food and separated bottles with tap water or 10% sucrose solution (at same time they had intermittent access to the sucrose solution). This independent group of rats was video-recorded (one continuous 60-min recording) to determine the effects of D2 dopamine receptor antagonism on the microstructure of feeding behavior.

### Histology

Following the behavioral experiments (intra-NAcS injections), rats with implanted cannulas were euthanized with an overdose of sodium pentobarbital and were decapitated. Brains were removed and post-fixed in 10% formalin; later they were sectioned at 200 μm in the coronal plane and were examined at low magnification to verify the position of the cannula. Brains in which the cannulas were not placed appropriately were excluded from the present report. All injections were confined to the NAcS and within 0.3 mm of its border.

### Data analysis

Data from body weight and energy intake (kcal, chow, and sucrose) during the experimental protocol (intermittent access to the sucrose solution) were analyzed by repeated measures two-way ANOVAs, considering exposition to the sucrose solution (no access, control; 2-h daily access, intermittent; 24-h daily access, *ad libitum*) and time (days) as the between-groups factor. The comparisons of energy intake (kcal, chow and sucrose) between estrous phases were analyzed by two-way ANOVAs, considering the estrous phase (estrous, diestrus) and groups (control, intermittent, *ad libitum*) as the between-groups factor. To calculate the significance of the differences in the operant test (response rate, breaking points) we entered two-way ANOVAs, considering the exposition to the sucrose solution (before the sucrose access, after the sucrose access) as the between-groups factor, and *control, intermittent*, and *ad libitum* as groups. Since data from behavioral characterization were not normally distributed (Shapiro–Wilk test) and/or variances were not equal, they were transformed by square root transformation, then outliers were identified and removed when appropriate according to Grubb's test, and finally we entered one-way ANOVAs to reveal differences between groups (control, intermittent, *ad libitum*, and intermittent + raclopride) in the microstructure of feeding behavior parameters (frequency, meal duration, inter-meal interval, latency, local eating rate, and durations of activity, and resting). Significant ANOVAs were followed by the Bonferroni's *post hoc* tests. In the final experiment, energy intake (from sucrose, chow, and total) was compared between groups (vehicle, raclopride 18 nM) using the paired student's *t*-test. The criterion for statistical significance was *p* < 0.05. Data were analyzed using GraphPad Prism Version 5.0, QuickCalcs (GraphPad Software, San Diego, CA, USA), and SPSS Version 17.0 (IBM, Chicago, IL, USA).

## Results

### Effects of intermittent access to the sucrose solution on body weight, energy intake, and fat accumulation

**Figure 1:**
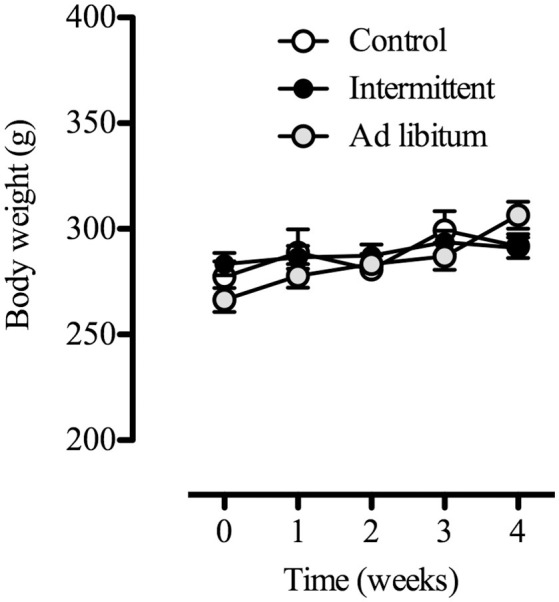
Body weight (g) of animals differentially exposed to the sucrose solution (10%): no access (control, *n* = 14), 2-h daily access (intermittent, *n* = 16), and 24-h daily access (*ad libitum, n* = 13) during the 28 days of the protocol. Data are expressed as means ± S.E.M.

**Figure 2:**
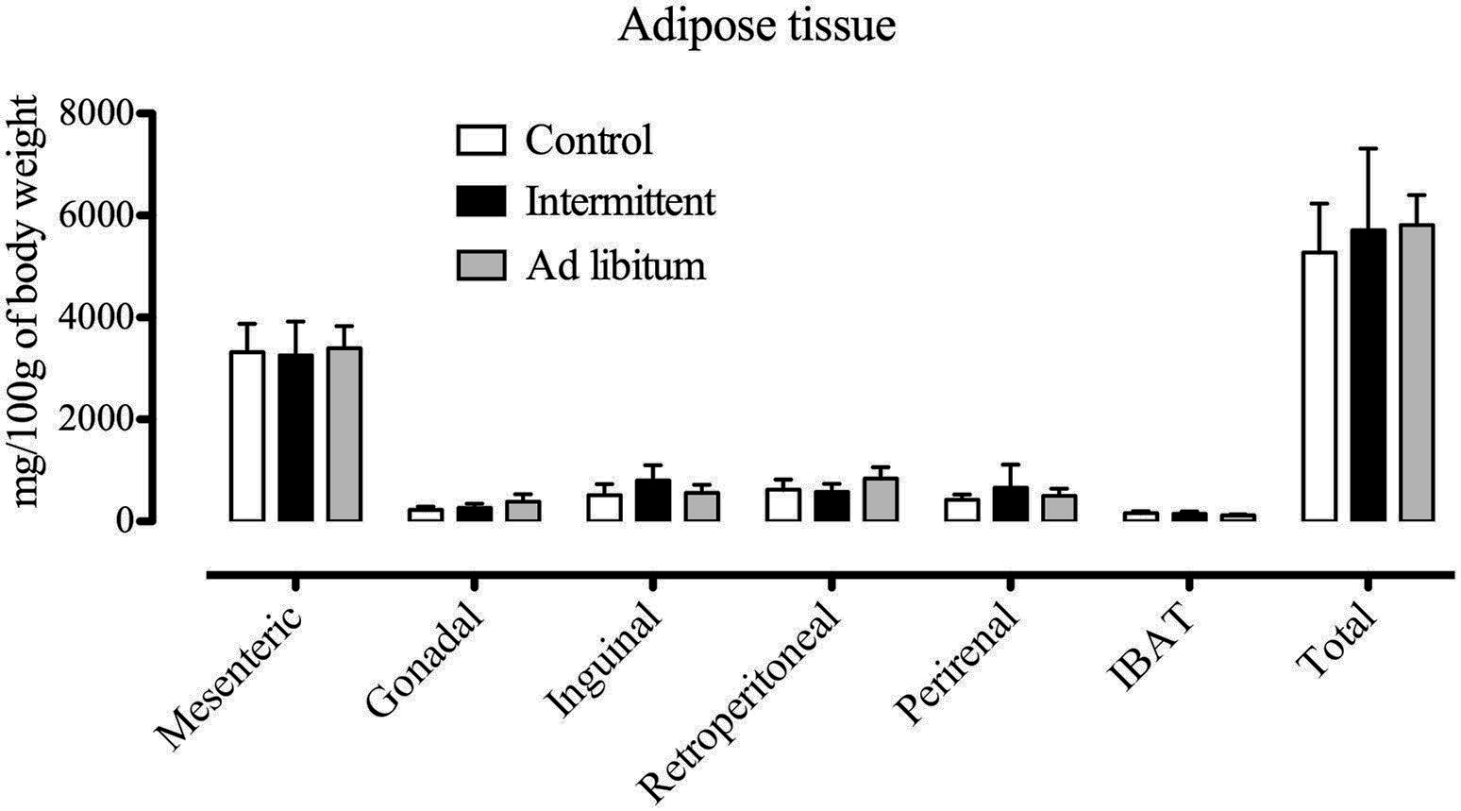
Measurements of adipose tissue in specific regions after the 28 days of exposition to the sucrose solution (*n* = 4 each group, control, intermittent, and *ad libitum*). Data are expressed as the normalization of mg of adipose tissue per 100 g of body weight. IBAT, interscapular brown adipose tissue (means ± S.E.M).

In order to characterize the feeding behavior microstructure of rats exposed to the intermittent access to the sucrose solution, we first evaluated changes in body weight, fat accumulation, and caloric intake from the standard diet (which was freely available in all groups, *n* = 13–16) and from the 10% sucrose solution. We found that all subjects continued gaining body weight during the protocol [time factor *F*_(4, 40)_ = 15.19; *p* < 0.01], without any significant difference among groups (Figure 1). Furthermore, accumulation of adipose tissue from different regions remained similar among groups (each group *n* = 4) (Figure 2).

**Figure 3:**
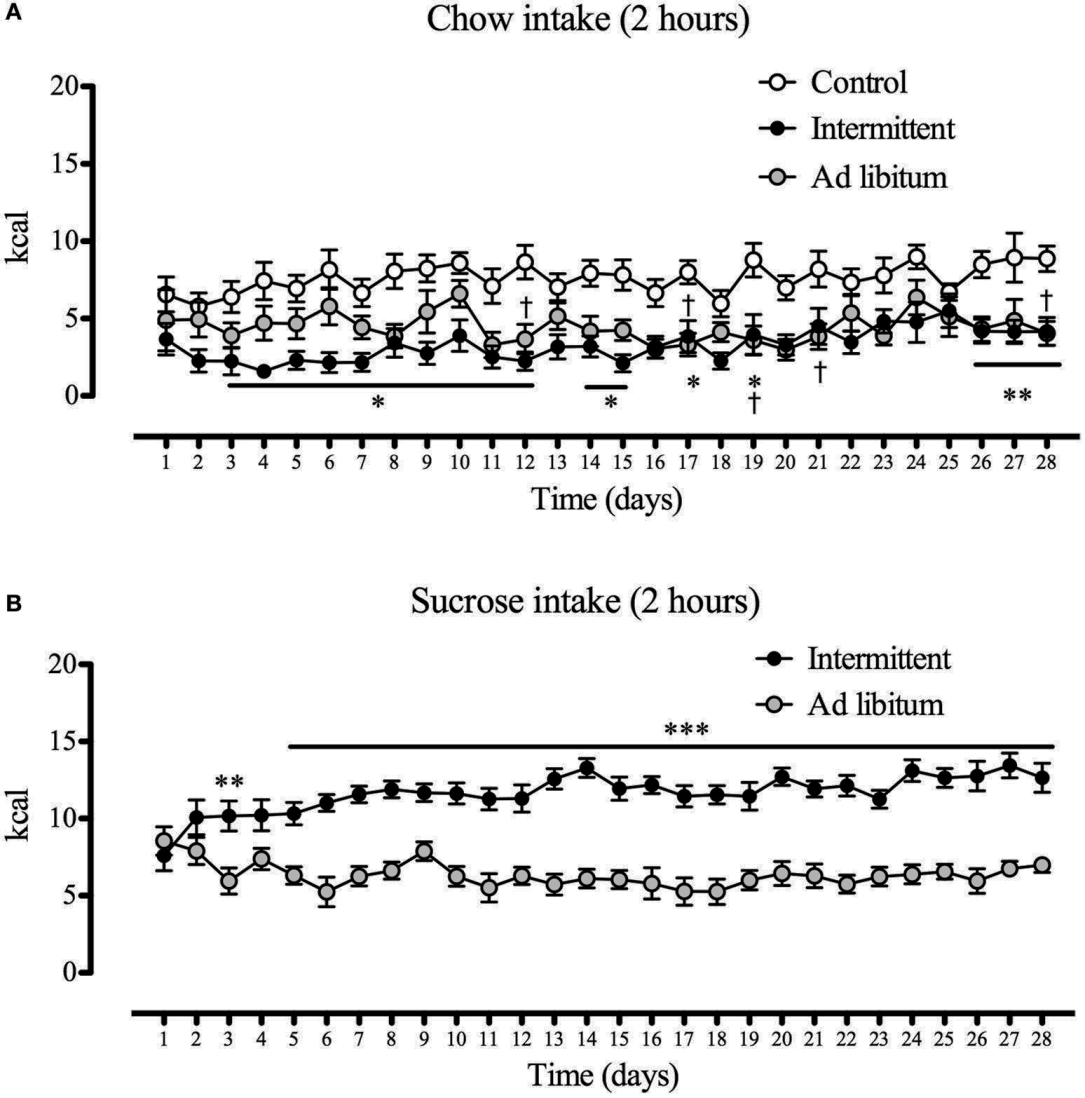
Daily energy intake from chow **(A)** and from the sucrose solution **(B)** of animals during the 28 days of exposition to the sucrose solution (control, *n* = 14; intermittent, *n* = 16; *ad libitum, n* = 13). Food and sucrose solution intake were measured in grams during a 2-h period, converted to kcal and expressed as means ± S.E.M. **(A)** *p < 0.05, **p < 0.01 intermittent group vs. control group; ^†^*p* < 0.05 *ad libitum* vs. control group. **(B)** **p < 0.01, ***p < 0.001 intermittent group vs. *ad libitum* group.

As far as energy intake is regarded, we found that the caloric consumption of the standard diet decreased significantly in both groups with access to sucrose solution (intermittent/*ad libitum*) [time factor *F*_(27, 40)_ = 1.80; *p* < 0.01; group factor *F*_(2, 40)_ = 33.57; *p* < 0.001] (Figure 3A). As expected, the energy consumed in the 2 h of access to the sucrose solution was significantly higher in subjects of the intermittent group [interaction *F*_(1, 27)_ = 3.56; *p* < 0.001; time factor *F*_(1, 27)_ = 1.99; *p* < 0.05; group factor *F*_(1, 40)_ = 89.89; *p* < 0.001]. Accordingly, subjects with intermittent access to sucrose solution consumed 50% more calories than subjects with *ad libitum* access to this solution from the 6th day to the end of the protocol (Figure 3B).

### Effects of estrous phase on sugar binging

**Figure 4:**
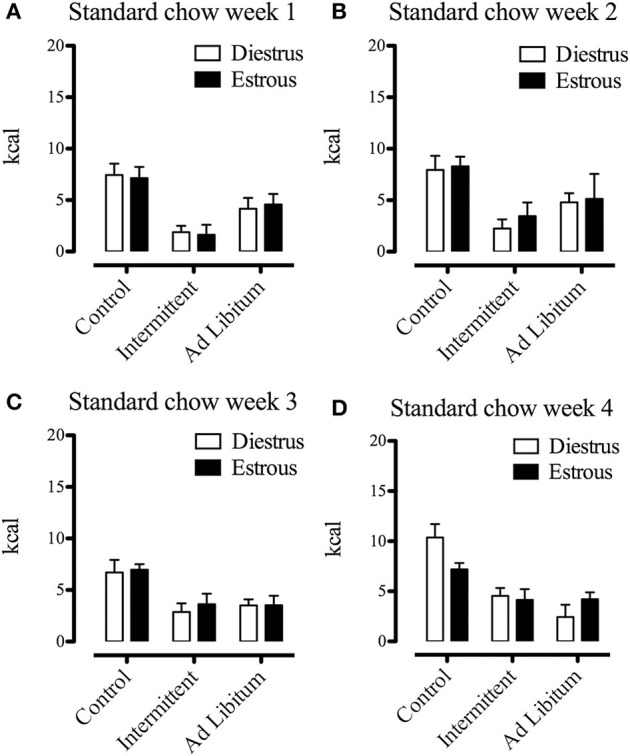
Effects of estrous phase on energy intake (from chow) of animals differentially exposed to the sucrose solution after 1 **(A)**, 2 **(B)**, 3 **(C)**, and 4 **(D)** weeks from the beginning of the protocol (*n* = 7–12). Food intake was measured in grams during a 2-h period, converted to kcal and expressed as means ± S.E.M.

**Figure 5:**
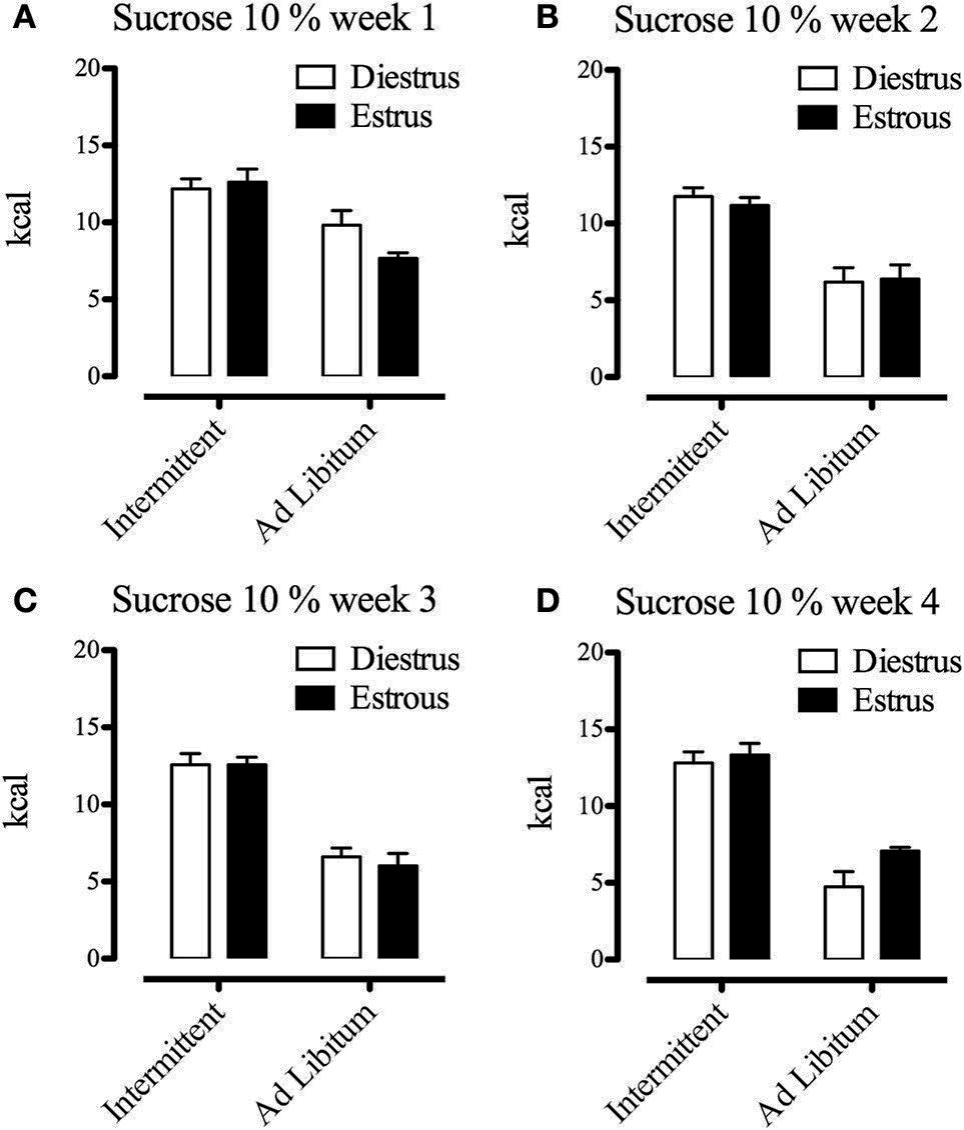
Effects of estrous phase on energy intake (form sucrose) of animals differentially exposed to the sucrose solution after 1 **(A)**, 2 **(B)**, 3 **(C)**, and 4 **(D)** weeks from the beginning of the protocol (*n* = 7–12). Sucrose solution intake was measured in grams during a 2-h period, converted to kcal and expressed as means ± S.E.M.

**Figure 6:**
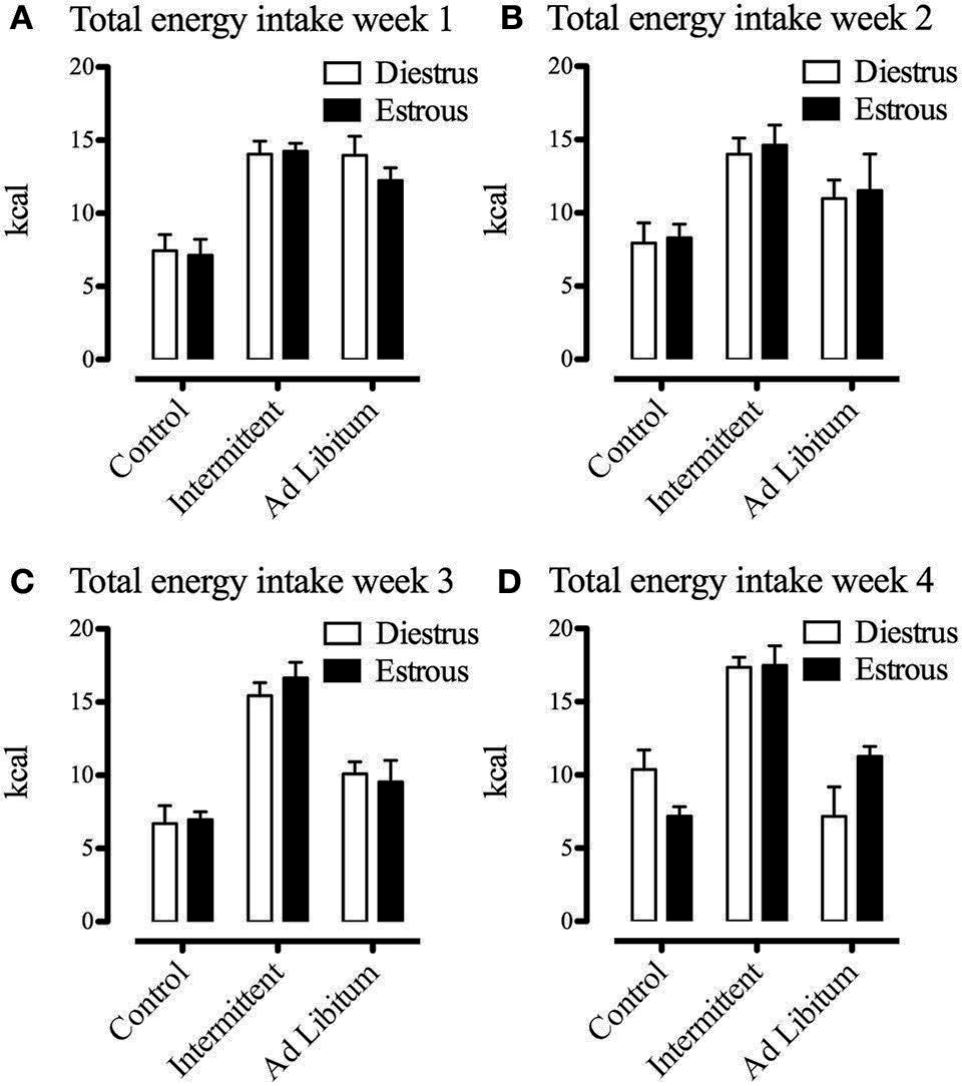
Effects of estrous phase on total energy intake (chow + sucrose) of animals differentially exposed to the sucrose solution after 1 **(A)**, 2 **(B)**, 3 **(C)**, and 4 **(D)** weeks from the beginning of the protocol (*n* = 7–12). Food and sucrose solution intake was measured in grams during a 2-h period, converted to kcal and expressed as means ± S.E.M.

It has been shown that feeding behavior is influenced by the ovarian hormone estradiol, producing phasic, and tonic inhibitory effects on food intake (Eckel, 2011). Since we used female Sprague Dawley rats, we considered the effects of estrous phase as a relevant factor and we evaluated whether this variable affected the sugar binging induced by intermittent access to the sucrose solution. Consequently, we compared energy intake (standard chow, sucrose solution, and total energy intake) at the end of each week of the protocol using the estrous phase (estrous vs. diestrus, *n* = 7–14) as between groups factor. Despite the fact that subjects exposed to the intermittent sucrose had significant increases of energy intake during the last 3 weeks of the protocol compared to the *ad libitum* group [week 2 *F*_(1, 33)_ = 40.80; *p* > 0.001; week 3 *F*_(1, 38)_ = 86.30; *p* > 0.001; week 4 *F*_(1, 36)_ = 93.26; *p* > 0.001], we did not find any difference of energy intake between estrous and diestrus phases (Figures 4–6). All rats had on average 6 estrous cycles during the intermittent access to sucrose protocol and the modality of access to sucrose solution (intermittent/*ad libitum*) did not modify the number of cycles (data not shown).

### Effects of intermittent access to sucrose solution on motivation for palatable food

**Figure 7:**
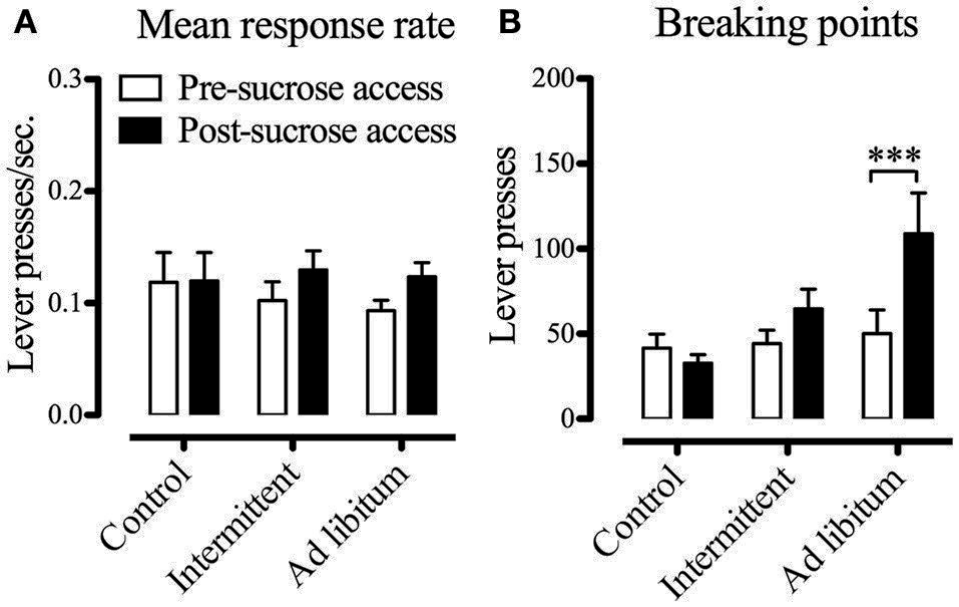
Mean response rate **(A)** and breaking points **(B)** of animals before and after the 28-day protocol (*n* = 5 each group, control, intermittent, and *ad libitum*). Data expressed as means ± S.E.M. ***p < 0.001 pre-sucrose access vs. post-sucrose access, *ad libitum* group.

Once we established that the model of intermittent access to the sucrose solution was not affected by the estrous phase, we evaluated in independent groups of rats (control, intermittent, and *ad libitum, n* = 5 each group) whether the protocol produced changes on the motivation for palatable food (chocolate flavor sucrose pellets). We hypothesized that chronic access to sucrose solution would increase BP, especially in the intermittent group, since it has been showed that chronic exposure to sucrose solutions produce a pattern of excessive intake (Colantuoni et al., 2001) induced by sensitization of the reward system (Hajnal et al., 2008). We compared the mean response rate (lever presses/time) and the BP of the subjects before and after they had access to the sucrose solution (training sessions vs. sessions after 4-week protocol, means of the last 3 sessions with <20% of variation). Unexpectedly, we found that the mean response rate was unaffected by the sucrose solution, and we did not find any difference among groups. Moreover, BP increased in those subjects with *ad libitum* access to the sucrose solution [*F*_(1, 12)_ = 11.98; *p* < 0.01], reflecting that in this particular condition the intermittent access to the sucrose solution did not change the motivational relevance of the palatable food (Figure 7).

### Effects of intermittent access to the sucrose solution and dopamine D2 blockade in the NAcS on the feeding behavior microstructure and on energy intake

In order to detect slight effects of the intermittent access to the sucrose solution not revealed by a simple measure of the amount of energy consumed, we characterized the microstructure of feeding behavior in animals with sugar-induced binging (*n* = 7) by computing the parameters of meal frequency, meal duration (s), inter-meal interval (s), latency (s), local eating rate (kcal/duration) and of activity and resting duration (Blundell, 1986). Furthermore, we tested the hypothesis that the administration of raclopride (D2 receptor antagonist, at a dose of 10-fold the K_i_, 18 nM, *n* = 4) would block the behavioral effects of intermittent access to the sucrose solution. Since binge eating by definition involves consumption of large amounts of food in a reduced period of time, we analyzed behavioral parameters in the first 60-min period although the intermittent group had 2-h daily access to the sucrose solution.

**Figure 8:**
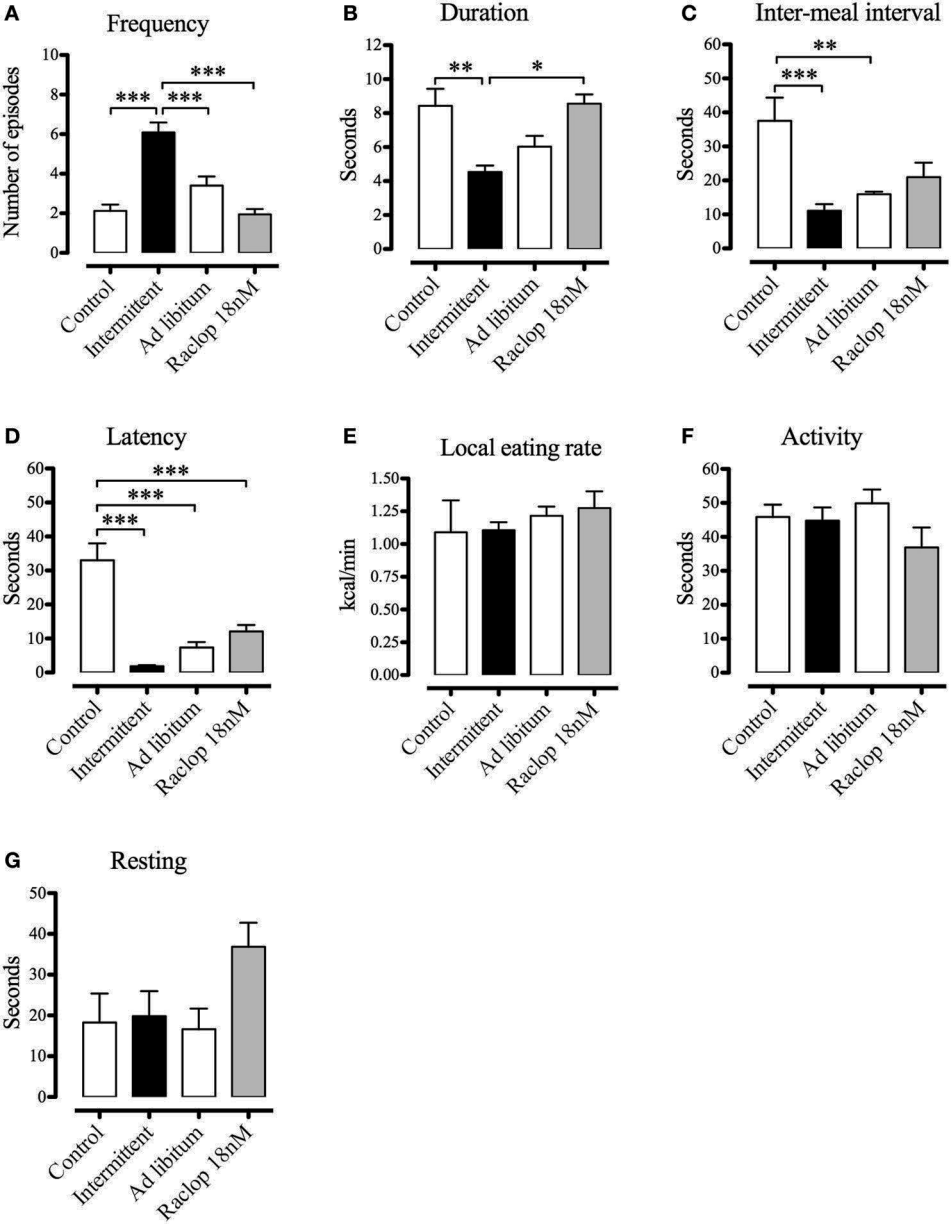
Microstructure of feeding behavior parameters, including meal frequency **(A)**, meal duration **(B)**, inter-meal interval **(C)**, latency **(D)**, local eating rate **(E)** and durations of activity **(F)**, and resting **(G)** of animals differentially exposed to the sucrose solution. A separate group of rats (*n* = 4) had intermittent access to the sucrose solution during 28 days and after recovery from stereotaxic surgery, raclopride (18 nM) was injected in the NAcS. Data expressed as means ± S.E.M. *p < 0.05, **p < 0.01, ***p < 0.001.

We found that intermittent access to the sucrose solution increased meal frequency [*F*_(3, 20)_ = 19.26; *p* < 0.001], and produced significant decreases of meal duration [*F*_(3, 19)_ = 8.19; *p* < 0.01], inter-meal intervals [*F*_(3, 19)_ = 8.76; *p* < 0.001], and latency [*F*_(3, 19)_ = 25.54; *p* < 0.001; Figures 8A–D], reflecting numerous consecutive short-duration episodes of eating that may be analogous to clinical observations of food craving, particularly for sugar and other carbohydrates, which can trigger or preserve impulsive eating (Avena et al., 2008b). Interestingly, pharmacological blockade of dopamine D2 receptors in the NAcS only prevented the effects of the intermittent access to the sucrose solution on meal frequency and duration, leaving the effects on inter-meal intervals and latency unaltered (Figures 8A–D). Access to the sucrose solution (irrespective of the condition or treatment) decreased inter-meal intervals and latency (Figures 8C,D), while the activity and resting durations, as well as the local eating rate, were unaffected by the experimental manipulations (Figures 8E–G).

**Figure 9:**
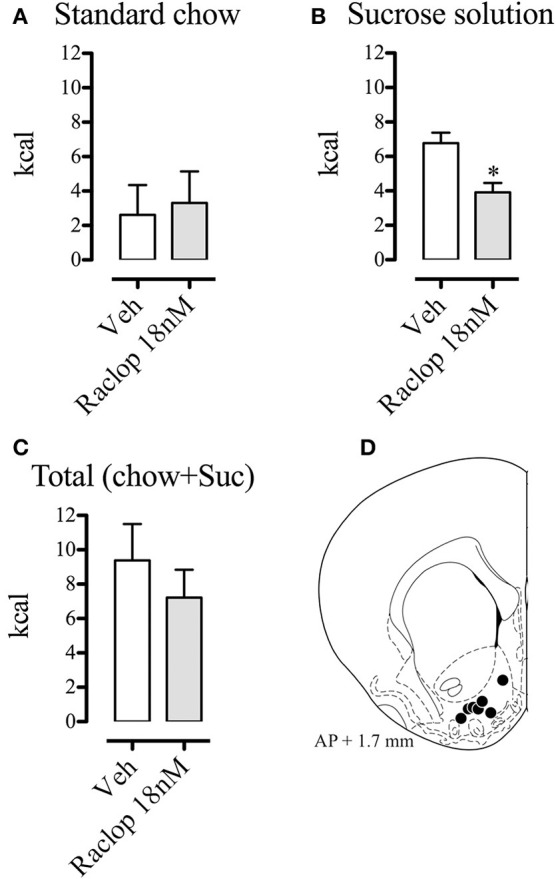
Effects of intra-NAcS injection of raclopride (18 nM) on energy intake from chow **(A)**, from the sucrose solution **(B)**, and total energy intake (chow + sucrose) **(C)**, after the 28 days of intermittent exposition to the sucrose solution (*n* = 8 each group). Food and sucrose solution intake were measured in grams during a 2-h period, converted to kcal and expressed as means ± S.E.M. Schematic illustration of the injection sites in the NAcS (black circles) (Paxinos and Watson, 1998) **(D)**. Veh, vehicle; Raclop, raclopride. *p < 0.05.

Finally, we compared the energy intake (chow, sucrose and total) of the subjects that had intermittent access to the sucrose solution and received intra-NAcS injections of vehicle or raclopride (18 nM) (Figure 9D). We found that blockade of dopamine D2 receptors specifically decreased the sucrose solution intake (*t* = 3.021; *p* < 0.05; Figure 9B). There were no differences in energy intake from standard chow, nor in the total energy intake (Figures 9A,C).

## Discussion

The present study aimed to characterize the microstructure of feeding behavior and motivation for palatable food in female Sprague-Dawley rats that expressed binge-eating behavior induced by chronic intermittent access to a sucrose solution, as well as to determine whether blockade of D2 dopamine receptors in the NAcS prevented the behavioral changes induced by this protocol. Here we have shown that rats intermittently exposed to the sucrose solution not only increased the energy intake (>50% from the 2nd to the 4th week of the protocol), but also expressed a particular binge-like feeding microstructure characterized by numerous consecutive short-duration episodes of eating. Furthermore, we found that intra-NAcS administration of raclopride prevented the changes in meal frequency and duration as well as it blocked the increase of sucrose consumption induced by intermittent protocol. As far as we know, this is the first report that characterized the microstructure of binge-like behavior in female rats chronically exposed to intermittent access to a sucrose solution, and that provided evidence that selective blockade of D2 receptors in the NAcS prevented some of the behavioral changes induced by intermittent sucrose.

Our results are consistent with the findings that limited access to palatable diets induces binge-type eating, independently of caloric restriction (Corwin, 2004). Although subjects in the intermittent group had free access to the standard chow, we found that rats consistently increased the energy intake when they had access to the sucrose solution. These findings are in agreement with clinical observations that triggering factors of binging are beyond the metabolic needs (APA, 2013) and that caloric restriction may be present but it is not a determinant factor (Corwin et al., 2011).

The effects of estrogens on food intake have been previously studied, and it is generally accepted that they have an inhibitory role (Eckel, 2011). Particularly with sugar binging rats, it has been shown that sucrose intake at the beginning of the dark photoperiod is not reduced during the estrous phase, as occurs with the standard chow (Calvez and Timofeeva, 2016). Correspondingly, we found that energy intake from the sucrose solution was not different between estrous phases, suggesting that sugar binging is not directly dependent of estrogens. Nevertheless, we did not observe the inhibitory effect of estrous phase on the consumption of the standard chow. This absence of a difference in chow consumption between estrous phases may be explained by the fact that we conducted the behavioral evaluation when the main food ingestion period had occurred (5 h after dark phase onset; Strubbe et al., 1986).

Several studies have proposed that intermittency and palatability of diets in binge-eating protocols are responsible for the persistent increases of dopamine release, a phenomenon that may explain the pattern of excessive intake (Colantuoni et al., 2001; Rada et al., 2005). According to these reports, changes in mesolimbic dopaminergic activity increase the susceptibility for addictive processes and predisposition to obesity induced by high-sugar palatable diets, altering the signaling of the D2 receptors in the NAcS and sensitizing the reward system (Hajnal et al., 2008). Despite those findings strongly suggesting that binging rats would increase motivation for palatable food, we find in the operant test that the motivation (breakpoints) for palatable rewards remained unaltered in the intermittent group. This finding indicates that changes induced by the intermittent exposure to sucrose solution did not increase the reinforcing value of palatable food, and that a different behavioral component of the rewarding/addictive process may be involved in the triggering of the binge-like behavior.

A reasonable explanation of this unexpected result is that impulsivity instead of motivational relevance of palatable food might drive binge-like behavior in our experimental conditions. In support of this hypothesis, several studies have shown that substance use disorders (voluntary ingestion of stimulants such as amphetamine, cocaine, or alcohol) coexist with impulsive behavior, alteration of mesolimbic dopaminergic transmission (Volkow et al., 2007; London, 2016), and particularly, with deficits in striatal dopamine D2-type receptors (Dalley et al., 2007). Furthermore, Johnson and Kenny (2010) found that knockdown of striatal D2 receptors in the dorsolateral striatum causes compulsive-like feeding behavior (consumption of food resistant to disruption by an aversive conditioned stimulus) in rats with extended access to palatable high-fat food. Considering that impulsivity are among the core symptoms of addictions (Tang et al., 2015), and that it consistently predicts the development of addiction to stimulants (Dalley et al., 2007; Perry and Carroll, 2008) as well as the susceptibility to the addictive properties of highly palatable foods (Velázquez-Sánchez et al., 2014), our observation of numerous consecutive short-duration episodes of eating in rats intermittently exposed the sucrose is comparable to impulsive eating, suggesting that intermittency of sugar intake induced binge-type microstructure and contributed to the deregulation of sugar intake via the increase of impulsivity. Further investigations with systematic and valid procedures are needed to characterize the exact nature of the relationship between impulsivity and sugar binging.

On the other hand, our findings that selective blockade of D2 receptors in the NAcS prevented the increase of energy intake from the sucrose solution and some of the behavioral changes induced by intermittent sucrose are consistent with those that establish that disturbance of dopaminergic signaling explain the pattern of excessive intake and D2 receptors in the NAcS are involved in this phenomenon (Bello et al., 2002; Spangler et al., 2004; Hajnal et al., 2008). Nevertheless, Lardeux et al. (2015) showed compelling evidence that intermittent access to a palatable diet (cream–oil–sucrose emulsion) does not cause overconsumption dependent on mesolimbic dopamine, since they found that intra-accumbens (core and shell) injections of SCH23390 or raclopride (D1 and D2 antagonists, respectively) failed to prevent binging in rats. The discrepancy between these results may be explained considering methodological differences including sex, strain, and diet composition, since Lardeux et al., used male Long–Evans rats and a mixture of carbohydrates and fat to induce binge-like behavior. Especially sex of the subjects is relevant, for instance, it has been shown that female Wistar-Kyoto rats developed a more stable and significant binge-like behavior than male rats (Papacostas-Quintanilla et al., 2017); and mice expressing DRD2S and lacking DRD2L drink more ethanol than wild-type subjects (both sexes), and interestingly this genotype is associated with excessive intake of sucrose solution in female mice (Bulwa et al., 2011). Furthermore, it was reported that intra-NAcS administration of sulpiride dose-dependently attenuated reinstatement of drug seeking induced by a cocaine priming injection (Anderson et al., 2006). Taking these findings together, it is likely that the dopaminergic transmission mediated by D2 receptors in the NAcS partially constitutes a triggering factor for binging, predominantly in female subjects and with high-sugar diets.

It should be noted that our result that the sugar binging was independent of the estrous phase is controversial, since the prevalence of binge eating is higher in women than in men (Davis, 2015), and it has been shown that binge-like behavior is decreased in ovariectomyzed mice with 17β-estradiol replacement (Cao et al., 2014). Probably, the estrogens do not contribute directly to the triggering of binging; however, they have a permissive role in the development of the binging behavior. Accordingly, Babbs et al. (2015), showed that administration of 2-hydroxyestradiol (a metabolite of estradiol) to ovariectomyzed rats before the development of binging facilitates the expression and intensity of binge-type behavior, and even can induce binge-type behavior in male rats (Babbs et al., 2011). Consequently, estrogens may be necessary for the establishment of the binging, although in later stages of the binge-like behavior animal models, their lack of effects (Calvez and Timofeeva, 2016) facilitates binging (Babbs et al., 2015), or prevents (Cao et al., 2014) binging. These paradoxical findings should be addressed in further research considering clinical observations that decreased levels of circulating estrogens are associated with increased binging in patients with BED (Edler et al., 2007).

Several studies have shown that changes in D2 dopamine receptors signaling and expression in the NAcS are strongly related to sucrose overconsumption and binging (Bello et al., 2002; Spangler et al., 2004; Zhang et al., 2007; Hajnal et al., 2008), as well as to drug-induced relapse in animal models of addiction (Anderson et al., 2006). Our results also provide an advance in the discernment of the neurochemical substrates of binge-like behavior. We found that pharmacological blockade of D2 receptors in the NAcS prevented the increment of energy intake from sucrose as well as the observed increment in the frequency and the decrement of duration of episodes induced by intermittent access protocol, suggesting that alterations in behavioral patterns associated with binge-eating behavior depend in part on the dopaminergic transmission in the NAcS, and that the antagonism of dopamine D2 receptors may be considered for the development of treatment options when binge-like behavior is part of the symptomatology.

Despite the fact that *ad libitum* access to the sucrose solution failed to induce binge-type eating, we found that this condition increased motivation for palatable rewards. Unlike most addictive substances, sugar-rich foods show their addictive properties under specific conditions, and the neurobiological changes produced by palatable food and drugs of abuse in the reward system are qualitatively and quantitatively different (De Jong et al., 2016). Similarly, the triggering factors of binging in obese patients and in BED patients are different; likewise, overeating, obesity, and food addiction are related but completely different concepts (Davis et al., 2013). According to our results and findings in the literature, the increase in dopaminergic transmission that may anticipate binge-eating behavior induced by intermittent exposure to sucrose (Rada et al., 2005; Bello and Hajnal, 2010), as well as the hyposensitivity of the reward system (due to the lower dopaminergic activity in the circuit of reward) in obesity (Geiger et al., 2008, 2009; Leigh and Morris, 2018) converge in the alteration of the dopaminergic transmission mediated by dopamine D2 receptors (Zhang et al., 2007; Johnson and Kenny, 2010; Davis et al., 2012, 2013). Accordingly, the dysfunction of the D2 receptors may lead to compensation through overeating to elevate central dopamine release in the reward system (in both cases of obesity and BED); however, this system is less sensitive to general reward but remains responsive to palatable foods (Geiger et al., 2009; Leigh and Morris, 2018).

Although our study has implications on the relation between dopamine signaling mediated by D2 receptors in the reward system and the neurobiological and behavioral triggering factors of binging, these must be considered within the context of several limitations. First, our results were obtained with female rats and, although binging occurs predominantly in women, future research should include male rats. In addition, intermittent access to the sucrose solution was always provided at the same time of day, which resulted in programming of binge-type behavior at the halfway through the natural activity period of rats, while patients with binging usually present this condition without planning and during the night, when the natural period of activity has concluded. Finally, circadian variations of the estrogens were not considered in the present study. Thus, future studies should carefully control the experimental conditions to improve the validity of the model, including a more similar context to the prevailing situations when binging occurs.

## Conclusion

Binge-eating behavior is a core symptom in several eating pathologies and understanding of the neurobiological triggering factors of this condition had remained elusive. In this study, we investigated the motivation and the microstructure of feeding behavior in animals that expressed sugar-induced binging and evaluated the effects of blocking D2 receptors in the NAcS. We found that the feeding microstructure was characterized by numerous consecutive short-duration episodes of eating, and that intra-NAcS administration of raclopride prevented the changes in meal frequency and duration as well as it blocked the increase of energy intake from sucrose. In our study, binging-induced was not related to changes in body weight, fat accumulation or motivation for palatable food; and it was independent of the estrous phase. Finally, our findings suggest that treatment with D2 dopamine antagonists should be considered as a plausible therapeutic alternative to treat feeding pathology with binge-eating behavior.

## Author contributions

JS-O, JM-D, and RE-P: designed the study and wrote the protocol; JS-O, AM-C, FC-S, VL-A, and JT-J: contributed to the acquisition, analysis and interpretation of the data; RE-P: wrote the first draft, and all authors revised and approved the final version of the manuscript.

### Conflict of interest statement

The authors declare that the research was conducted in the absence of any commercial or financial relationships that could be construed as a potential conflict of interest.
