# Corrigendum: Intra-accumbens raclopride administration prevents behavioral changes induced by intermittent access to sucrose solution

**DOI:** 10.3389/fnins.2023.1163637

**Published:** 2023-03-13

**Authors:** Josué O. Suárez-Ortiz, Felipe Cortés-Salazar, Ariadna L. Malagón-Carrillo, Verónica E. López-Alonso, Juan M. Mancilla-Díaz, Juan G. Tejas-Juárez, Rodrigo E. Escartín-Pérez

**Affiliations:** ^1^Laboratory of Neurobiology of Eating, Facultad de Estudios Superiores Iztacala, Universidad Nacional Autónoma de México, Tlalnepantla, Mexico; ^2^División Académica Multidisciplinaria de Comalcalco, Universidad Juárez Autónoma de Tabasco, Tabasco, Mexico

**Keywords:** binge-type eating, dopamine d2 receptors, microstructure, raclopride, intermittent sucrose, nucleus accumbens shell

In the published article, there was an error in the Funding statement. [The Funding statement indicated that the study was funded by UNAM DGAPA grants IN224214 and IN217117. We omitted CONACYT fellowship 345359 to JS-O]. The correct Funding statement appears below:

